# Supplementary material for: Remote physical function testing in older adults: a mixed methods study exploring test reliability, feasibility, and perceptions of participants and assessors
Source: PLoS One. 2025 Sep 19;20(9):e0332691. doi: 10.1371/journal.pone.0332691 (PMC12449032; doi:10.1371/journal.pone.0332691)
Supplement: S1 File — Interview schedule. (DOCX) [file pone.0332691.s001.docx]

**Supporting information file 1**: Interview schedule

*Participant interviews*

| **Prompt/main question** | **Follow-up questions** |
| --- | --- |
| General/intro questions | |
| What are your general impressions about participating in this study? |  |
| Barriers to testing | |
| What made the tests more challenging/harder to complete within your home? |  |
| What made the tests less challenging/easier to complete within your home? |  |
| Facilitators of testing | |
| Could anything be modified about the tests to make them easier to complete within your home? | If yes, what are they? |
| Do you think there are any benefits to performing these tests at home regularly? | If yes, what are they? |
| Acceptability of testing | |
| How did you feel while (during) performing the tests within your home? | Why did you feel this way?  Did this change during the study? |
| How did you feel after performing the tests within your home? | Why did you feel this way?  Did this change during the study? |
| Did you feel capable when completing the tests at home? | Why did you feel this way?  Did this change during the study? |
| Feasibility of testing |  |
| Do you think you would complete similar tests regularly within your home? |  |
| Do you have any concerns about completing similar tests within your home? |  |

*Assessor interviews*

| **Prompt/main question** | **Follow-up questions** |
| --- | --- |
| General/intro questions | |
| Could you describe your level of experience with conducting physical function tests or other forms of assessment in-person or remotely? | Any experience with older adults specifically?  Ask about remote assessments in particular if not covered. |
| What were your general impressions about your role as a practitioner or research assistant in this study? | Would you say your impressions were overall positive or negative? |
| Barriers to testing | |
| As a practitioner or research assistant, was there anything that made the tests more challenging to administer with older adults within their home? | If yes, what were they? |
| Was there anything that made the tests less challenging to administer with older adults within their home? | If yes, what were they? |
| Facilitators of testing | |
| Could anything be modified about the testing protocols to make these easier to administer with older adults within their home? | If yes, what were they? |
| As a practitioner or research assistant, do you think there are any benefits to being able to administer the physical function tests with older adults within their homes? | If yes, what were they? |
| Acceptability of testing | |
| How would you describe your sense of capability when administering the tests with older adults within their home? | Why did you feel this way?  Did this change during the study? |
| How do you think the results of the physical function tests would compare to the same tests conducted in person? | Why did you feel this way? |
| Did you find any aspects of administering the tests remotely with older adults to be burdensome or difficult? | If yes, what were they?  Did this change during the study? |
| During the testing sessions, did you have any concerns about the older adults completing the tests at home? | If yes, what were they?  Did this change during the study? |
| Did you feel the participants were able to understand how to complete the tests? |  |
| Did you feel the participants were able to understand why they were doing the tests? |  |
| Feasibility of testing | |
| Do you think the older adults you conducted the testing with would complete similar tests regularly within their home? |  |
| If you had the choice between administering the tests with an older adult remotely or face-to-face, which would you prefer and why? |  |
