# Supplementary material for: Remote physical function testing in older adults: a mixed methods study exploring test reliability, feasibility, and perceptions of participants and assessors
Source: PLoS One. 2025 Sep 19;20(9):e0332691. doi: 10.1371/journal.pone.0332691 (PMC12449032; doi:10.1371/journal.pone.0332691)
Supplement: S2 File — Bias and limits of agreement values for all physical function assessments between tests 1 versus 2 and between tests 2 versus 3. (DOCX) [file pone.0332691.s002.docx]

**Supporting information file 2**: Bias and limits of agreement values for all physical function assessments between tests 1 vs. 2 and between tests 2 vs. 3.

| **Test** | **Trial comparison** | **Bias** | **SD of bias** | **95% Limits of Agreement** |
| --- | --- | --- | --- | --- |
| 5-STS | 1 vs. 2 | 0.91 | 1.79 | -2.59, 4.41 |
|  | 2 vs. 3 | 0.00 | 1.60 | -3.14, 3.13 |
| 30-STS | 1 vs. 2 | -0.63 | 1.46 | -3.50, 2.23 |
|  | 2 vs. 3 | 0.11 | 1.10 | -2.05, 2.26 |
| FSST | 1 vs. 2 | 1.21 | 2.30 | -3.30, 5.73 |
|  | 2 vs. 3 | 0.24 | 1.80 | -3.30, 3.77 |
| Gait 2.44 m usual speed | 1 vs. 2 | -0.09 | 0.18 | -0.44, 0.26 |
|  | 2 vs. 3 | 0.02 | 0.13 | -0.24, 0.27 |
| Gait 2.44 m fastest speed | 1 vs. 2 | -0.09 | 0.17 | -0.42, 0.25 |
|  | 2 vs. 3 | -0.03 | 0.17 | -0.35, 0.30 |
| Gait 4 m usual speed | 1 vs. 2 | -0.05 | 0.12 | -0.29, 0.19 |
|  | 2 vs. 3 | -0.07 | 0.15 | -0.36, 0.22 |
| Gait 4 m fastest speed | 1 vs. 2 | -0.09 | 0.13 | -0.35, 0.17 |
|  | 2 vs. 3 | -0.04 | 0.25 | -0.52, 0.44 |
| Single-leg balance | 1 vs. 2 | 0.92 | 5.09 | -9.06, 10.89 |
|  | 2 vs. 3 | -0.20 | 3.16 | -6.40, 6.00 |
| SPPB total score | 1 vs. 2 | -0.79 | 1.13 | -3.01, 1.43 |
|  | 2 vs. 3 | -0.05 | 1.03 | -2.06, 1.96 |
