# Supplementary figures and images for: Remote physical function testing in older adults: a mixed methods study exploring test reliability, feasibility, and perceptions of participants and assessors
Source: PLoS One. 2025 Sep 19;20(9):e0332691. doi: 10.1371/journal.pone.0332691 (PMC12449032; doi:10.1371/journal.pone.0332691)

**Supporting information file 3**: Frequencies of total SPPB score across the participant cohort.


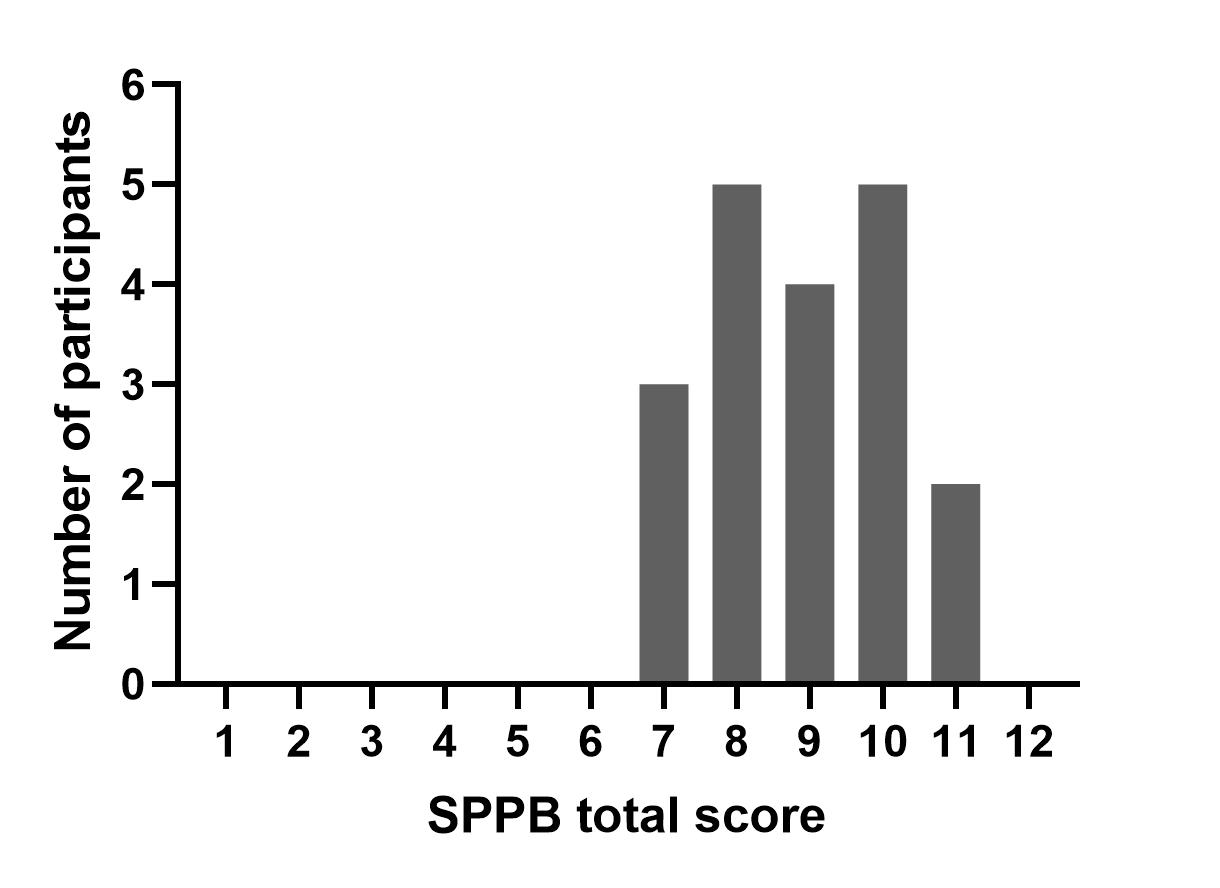

Supplement: S File — Frequencies of total SPPB score across the participant cohort. (DOCX) [file pone.0332691.s003.docx]
